# Isobaric Tags for Relative and Absolute Quantitation-Based Proteomics Analysis Revealed Proteins Involved in Drought Response during the Germination Stage in Faba Bean

**DOI:** 10.3390/metabo14030175

**Published:** 2024-03-21

**Authors:** Changyan Liu, Fangwen Yang, Li Li, Xuesong Han, Hongwei Chen, Aihua Sha, Chunhai Jiao

**Affiliations:** 1 Institute of Food Crops, Hubei Academy of Agricultural Sciences, Wuhan 430063, China; liucy0602@163.com (C.L.); hslili07@163.com (L.L.); hxs.1204@163.com (X.H.); hwchen25@126.com (H.C.); 2 Key Laboratory of Crop Molecular Breeding, Ministry of Agriculture and Rural Affairs, Wuhan 430063, China; 3 Hubei Key Laboratory of Food Crop Germplasm and Genetic Improvement, Wuhan 430063, China; 4 R&D Department, Genscript Biotech, Nanjing 211100, China; fangwen_yang@163.com; 5 MARA Key Laboratory of Sustainable Crop Production in the Middle Reaches of the Yangtze River (Co-Construction by Ministry and Province), Jingzhou 434025, China; 6 Engineering Research Center of Ecology and Agricultural Use of Wetland of Ministry of Education, Jingzhou 434025, China; 7 College of Agriculture, Yangtze University, Jingzhou 434025, China

**Keywords:** faba bean, germination, drought, iTRAQ, histone protein, SOD

## Abstract

The faba bean, a significant cool-season edible legume crop, is susceptible to drought during the germination stage. Research regarding the genetic regulation of drought tolerance throughout this stage in the faba bean is limited. The differentially expressed proteins (DEPs) in faba beans between the drought-tolerant variety C105 and the drought-sensitive variant E1 during seed germination were identified in this work, accomplished through isobaric tags for relative and absolute quantitation (iTRAQ) analysis. A total of 3827 proteins were identified in the two varieties of germinating seeds. Compared to those of variety E1, an increase in 108 DEPs and a decrease in 61 DEPs were observed in variety C105 under drought. Conversely, in the control group, variety C105 showed 108 significantly upregulated DEPs and 55 significantly downregulated DEPs. GO and KEGG analyses showed that the DEPs associated with glutathione metabolism and protein processing demonstrated significant increases in response to drought stress. Protein–protein interaction (PPI) analysis unveiled three closely connected functional modules of protein translation, DNA replication, and post-translational modification, originating from 22 DEPs derived from the germination period of two varieties under drought stress. To verify the proteomic function, we selected three differentially expressed protein coding genes, which were overexpressed or silenced in tobacco, thereby enhancing the drought resistance of tobacco. This was accompanied via altered levels of superoxide dismutase or peroxidase in transgenic plants under drought stress. The possible mechanism for drought tolerance in germinating seeds of faba bean involves increasing protein translation, decreasing DNA replication, and modifying chromatin. These findings offer invaluable insights into the reaction mechanism in response to drought stress in faba beans. The identified DEPs could be utilized in faba bean breeding initiatives to manage drought.

## 1. Introduction

Drought, amplified by global climate change, detrimentally affects plant growth and productivity by disrupting plant physiology, relationships with water, and photosynthesis rates, and by reducing chlorophyll, carotenoids content, and stomatal conductance [1]. Every development stage of crop plants, from seed germination to seed and fruit development, is sensitive to dehydration [2]. However, seed germination is primarily impacted by drought stress [3], compromising the uniformity and rate of seed germination, and consequently affecting the establishment, growth, and productivity of crops in drought conditions [4].

Regarded as a vital legume consumed by humans and animals and used for green-manure and soil improvement through symbiotic nitrogen fixation, the faba bean (*Vicia faba* L.) is widely cultivated worldwide. Its annual production has reached approximately 4.5 million tons, being predominantly produced in China, Ethiopia, Egypt, Australia, the United Kingdom, and France (FAOSTAT, http://www.fao.org/statistics/en, accessed on 13 March 2019). The faba bean’s growth requires a substantial amount of water, making it more drought-sensitive than other field crops [5,6]. This is particularly evident in the winter faba bean, which struggles to germinate when sown in the relatively dry autumn of south China and the Yangtze River region. Consequently, it is crucial to understand the molecular basis of drought tolerance during seed germination to breed drought-resistant cultivars adapted to water-limited environments. The faba bean genome, with its substantial size (~13 Gb) and complexity (over 85% repetitive DNA), complicates gene cloning through mapping-based approaches, despite the availability of published reference genome information [7]. Consequently, high-throughput methodologies such as transcriptome and proteome analysis present valuable alternatives for enriching genomic resources.

Numerous studies have been conducted to explore the genetic regulation of late-stage drought tolerance in the faba bean. Investigations have revealed a total of 35 possible drought-stress-related expressed sequence tags (ESTs) and 36,834 and 35,510 unigenes differentially expressed under drought stress in the seedling leaf and root, respectively, during the vegetative and flowering stages [8,9]. Fifty differentially expressed proteins (DEPs) were detected in the seedling of the drought-tolerant faba bean cultivar “Ga da dou” in response to drought stress [5]. Moreover, 9126 differentially expressed genes (DEGs) were identified in the leaves of faba bean Qinghai 13 under soil drought [10]. The quantitative trait loci (QTL) and candidate genes affecting drought adaptation-related stomatal morphology were identified using 188 polymorphic single-nucleotide polymorphisms (SNPs). [11]. The calcium-dependent protein kinase VfCPK1 is known to be induced in leaves by drought and abscisic acid [12]. However, the effect of drought on seed germination in the faba bean has received limited attention.

Several studies have endeavored to understand the genetic underpinnings of drought tolerance during seed germination [4,13,14,15,16,17,18,19]. Yet, the genetic regulation of drought tolerance during this phase remains largely elusive. In this study, we conducted proteomic analysis of two extreme-drought-tolerant varieties (C105 and E1) obtained through screening. It was found that proteins related to DNA replication, translation, and post-translational modifications were differentially accumulated, forming a regulatory module under drought stress. These findings offer fundamental insight into the genetic regulation of drought stress and form a basis for breeding drought-tolerant faba bean during seed germination.

## 2. Materials and Methods

### 2.1. Plant Materials

The faba bean varieties C105 and E1 were verified to be tolerant and sensitive to drought stress in previous study, respectively [20]. To conduct iTRAQ analysis, the seeds of C105 and E1 were surface-sterilized with 10% hypochlorite, washed with sterile water, soaked with 10% mannitol solution, and germinated in a growth chamber, as described by Wei et al., 2023 [20]. Distilled-water-treated seeds were used as controls. After treating for 64 h, we removed the cotyledons of fava beans and only retained the embryo, which was immediately frozen in liquid nitrogen for iTRAQ and qRT-PCR analyses.

### 2.2. Protein Extraction and Trypsin Digestion

By grinding the sample into powder using liquid nitrogen and subsequently transferring it into a 10 mL centrifuge tube, we prepared 3 copies of each sample as biological replicates. Following this, a lysis buffer consisting of 1% Triton-100, 8 M urea, 1% protease inhibitor, and 10 mM dithiothreitol cocktail was added to the powder. The mixture was then sonicated thrice on ice using a high-intensity ultrasonic processor. Any remaining debris was eliminated by centrifugation at 4 °C for 10 min at a speed of 20 ×g to eliminate any residual debris. The protein was precipitated with cold 20% TCA for 2 h at −20 °C. After centrifugation under similar conditions, the supernatant was discarded. We washed the remaining sediment three times with cold air, then dissolved the protein again in 8 M urea, and finally measured the protein concentration using a BCA assay kit (Yuanxing, Shanghai, China).

We digested the protein sample with 5 mM dithiothreitol at 56 °C for 30 min, which we then alkylated with 11 mM iodoacetamide at room temperature in the dark for 15 min. Then, in order to reduce the urea concentration to below 2 M, the protein sample was diluted with 100 mM TEAB. Finally, we added trypsin in a 1:50 mass ratio of trypsin to protein for the first overnight digestion, and we added trypsin in a 1:100 mass ratio of trypsin to protein for the second 4 h digestion.

### 2.3. iTRAQ Labeling and LC-MS/MS Analysis

After digestion with trypsin, the peptide was desalinated using a Strata X C18 SPE column. Subsequently, drying was carried out through the vacuum method. The peptide was reconstructed in 0.5 M TEAB.

The trypsin-digested peptides were dissolved in 0.1% formic acid, designated as solvent A, and were immediately applied to a self-made reversed-phase analytical column with dimensions of 15 cm in length and 75 μm in internal diameter. Over a duration of 26 min, the gradient was gradually adjusted from 6% to 23% of solvent B, which consisted of 0.1% formic acid dissolved in 98% acetonitrile. Subsequently, the percentage of solvent B was increased to 35% in 8 min and then rapidly rose to 80% in 3 min. The final 3 min involved maintenance at an 80% solvent B concentration.

The peptides were subjected to an NSI source followed by tandem mass spectrometry (MS/MS) in Q ExactiveTM Plus (Thermo Fisher Scientific, Waltham, MA, USA) coupled online to UPLC. The electrospray voltage applied was 2.0 kV. The *m*/*z* scan range was set from 350 to 1800 for a full scan, and intact peptides were detected in an Orbitrap at a resolution of 70,000. Peptides were then selected for MS/MS using an NCE setting of 28, and the fragments were detected in the Orbitrap at a resolution of 17,500. A data-dependent procedure alternated between one MS scan and 20 MS/MS scans with a 15.0 s dynamic exclusion. The automatic gain control (AGC) was set at 5E4, and the fixed first mass was set to 100 *m*/*z*.

### 2.4. Database Search

The resultant MS/MS data were processed using the Maxquant search engine (version 1.5.2.8). Tandem mass spectra were searched against the faba bean transcriptome database [DDBJ/ENA/GenBank] available at the National Center for Biotechnology Information (https://www.ncbi.nlm.nih.gov (GISP00000000) accessed on 25 February 2022), which was concatenated with a reverse decoy database. Trypsin/P was specified as the cleavage enzyme, with up to four missing cleavages allowed. The mass tolerance for precursor ions was set at 20 ppm for the first search and 5 ppm for the main search, while the mass tolerance for fragment ions was set at 0.02 Da. Carbamidomethyl on Cys was specified as a fixed modification, while acetylation modification and oxidation on Met were specified as variable modifications. The false discovery rate (FDR) was adjusted to less than 1%, and the minimum score for modified peptides was set to more than 40.

### 2.5. Gene Ontology Analysis Enrichment

Proteins were categorized based on GO annotations into three groups: biological process, cellular compartment, and molecular function. In each category, a two-tailed Fisher’s exact test was used to examine the enrichment of differentially expressed proteins against all identified proteins. A GO term with a corrected *p*-value of less than 0.05 was deemed significant.

### 2.6. Pathway Analysis Enrichment

The KEGG database was utilized to pinpoint enriched pathways. This was achieved through a two-tailed Fisher’s exact test, which examined the enrichment of differentially expressed proteins against all proteins identified. Pathways with a corrected *p*-value of less than 0.05 were deemed significant. These pathways were then organized into hierarchical categories in accordance with the KEGG website.

### 2.7. Protein–Protein Interaction Network

The database accession or sequence for all differentially expressed proteins was cross-referenced with the STRING database version 10.1 for protein–protein interactions. Only interactions within the searched dataset were selected, thereby excluding any external candidates. A metric used by STRING, termed “confidence score”, was used to define the confidence of interaction; all interactions with a confidence score of 0.7 or higher (high confidence) were retrieved. The interaction network from STRING was visualized using the “networkD3” package in R.

### 2.8. RNA Extraction and qRT-PCR

We extracted total RNA using TRIZOL reagent, which was treated with DNase (Invitrogen, Carlsbad, CA, USA) without RNase. We used a RevertAid™ First Strand cDNA synthesis kit (Thermo Fisher Scientific, Waltham, MA, USA) for reverse transcription of the first cDNA strand. qRT-PCR was carried out using gene-specific primers (Table S5) in a CFX96™ Real-Time PCR Detection System (Bio-Rad, Hercules, CA, USA). NADH dehydrogenase subunit 4 (*NADHD4*) of faba bean was used as the internal control. Each reaction comprised 10 μL volumes containing 5 μL of SYBR Premix Ex Taq™ (TAKARA, Beijing, China), 0.3 μL of each of forward and reverse primers (10 μM), 2 μL of cDNA template, and 2.4 μL of ddH_2_O. We used the following program to perform a triplicate reaction on each gene: 40 cycles at 95 °C for 30 s, 95 °C for 5 s, and 57–60 °C for 30 s. We used the melting curves to confirm the specificity of primers. We used the 2^−ΔΔCT^ method to calculate relative gene expression levels.

### 2.9. Silencing, Ectopic Expression of Targets, and RT-PCR

Four-week-old *N. benthamiana* plants, grown under a controlled environment (24 °C, 16 h/8 h light/dark, 100 μM m^−2^ s^−1^ white light), were used for target silencing or ectopic expression. Target fragments or full coding sequences (CDS) were amplified and inserted into the PVX-LIC vector as delineated by Han et al., 2021, with sequencing confirming the results. These constructs were subsequently introduced into *Agrobacterium tumefaciens* GV3101 through the freeze–thaw method and then into *N. benthamiana* via the infiltration method [19]. The empty PVX-LIC vector served as a negative control when introduced into tobacco. The experiment was carried out thrice, with a minimum of five plants used for each construct. Seven days postinfiltration, the uninoculated leaves (newly developing) were harvested for RNA extraction and RT-PCR analysis, and water was withheld to induce drought stress. The phenotype was photographed 10 days after water withholding. Gene-specific primers were utilized for the RT-PCR, and actin was employed to normalize the reaction, as described by Han et al., 2021 [19].

### 2.10. Physiological Parameter Measurements and Statistical Analysis

Newly developed, uninoculated leaves that had undergone water withholding for seven days were harvested for superoxide dismutase (SOD) and peroxidase (POD) activity analysis, as well as for proline and MDA content examinations, following the methods outlined by Han et al., 2021 [19]. Data analysis was conducted using Microsoft Excel 2016 and SPSS 16.0. One-way ANOVA was used to evaluate statistical significance, and Tukey’s multiple comparison test was employed to compare differences at a 0.05 significance level.

## 3. Result

### 3.1. Quantitative Proteomic Analysis

**Figure 1:**
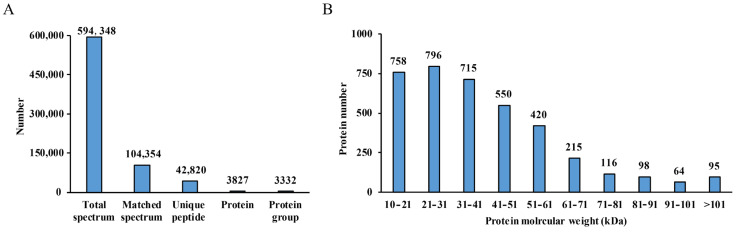
(**A**) Basic statistics of protein information for iTRAQ analysis. (**B**) The molecular weight distribution of proteins.

In this study, the proteomic analysis was conducted using the previously identified drought-tolerant variety C105 and the drought-sensitive variety E1 [20]. The seeds of C105 and E1 were treated with a mannitol (MA) solution, designated as C_T and E_T, respectively. Deionized water was used as a control for both seed types, referred to as C_CK and E_CK, respectively. After a treatment period of 64 h, the seeds were collected for protein profile analysis using isobaric tags for relative and absolute quantitation (iTRAQ) technology. The analysis yielded a total of 594,348 spectra, with 10,4354 matches. Following data merging, 42,820 unique peptides and 3827 proteins were observed, grouped into 3332 categories (Figure 1A). The majority of proteins, accounting for 84.63%, had a molecular weight ranging from 10 to 61 kDa. The remaining proteins had a molecular weight between 61 and 171 kDa (Figure 1B).

### 3.2. Identification of DEPs

**Figure 2:**
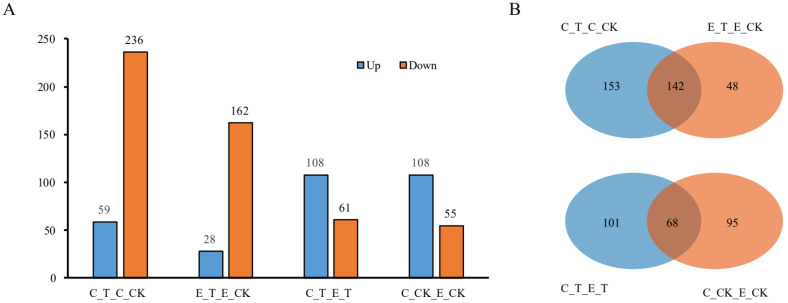
Quantitative and Venn analysis for proteins in the two faba bean varieties under different treatments. (**A**) Quantitative analysis of the proteins between drought treatment and control; (**B**) Venn analysis of proteins in faba bean with different treatments. The seeds of C105 and E1 were treated with a mannitol (MA) solution, designated as C_T and E_T, respectively. Deionized water was used as a control for both seed types, referred to as C_CK and E_CK, respectively.

Drought-responsive proteins were identified by comparing protein abundance between the control and MA-treated samples using iTRAQ data. The defined criteria for identifying DEPs were a fold change ratio >1.50 or <0.60 and a *p*-value < 0.05. These criteria were applied to compare C_T and C_CK, E_T and E_CK, C_T and E_T, and C_CK and E_CK. The four pairwise comparisons resulted in the identification of 295, 190, 169, and 163 DEPs, respectively (Table S1, Figure 2A). Among these DEPs, 59, 28, 108, and 108 were upregulated, while 236, 162, 61, and 55 were downregulated, respectively. In both the C105 and E1 varieties, more than 80% of DEPs were downregulated under drought stress conditions when compared to those under control conditions (Table S1). This suggested that drought stress inhibited the accumulation of most proteins involved in seed germination. Meanwhile, 142 DEPs were found to be responsive to drought stress in both C105 and E1 (Figure 2B), indicating that these proteins may play fundamental roles in drought stress signaling. Of the 169 and 163 DEPs identified in the C_T and E_T, and C_CK and E_CK comparisons, respectively, 68 were present in both (Figure 2B). This suggests that these DEPs may be variety-specific proteins that are not influenced by drought stress.

### 3.3. Functional Annotation of DEPs

**Figure 3:**
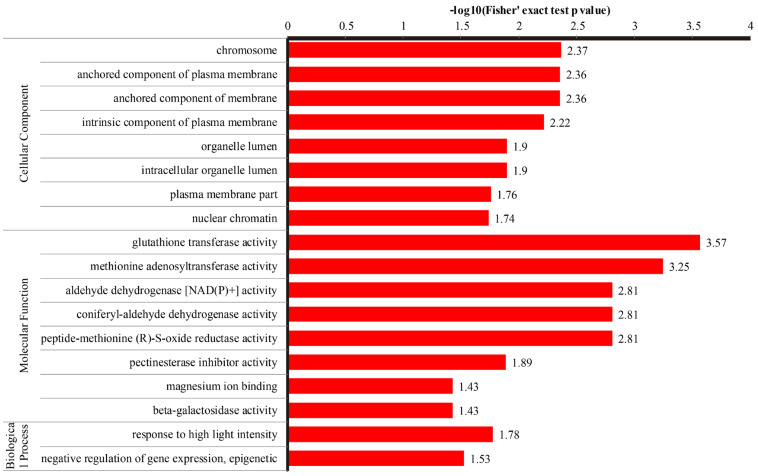
The Gene Ontology (GO) functional annotation of DEPs between C_T and E_T.

**Figure 4:**
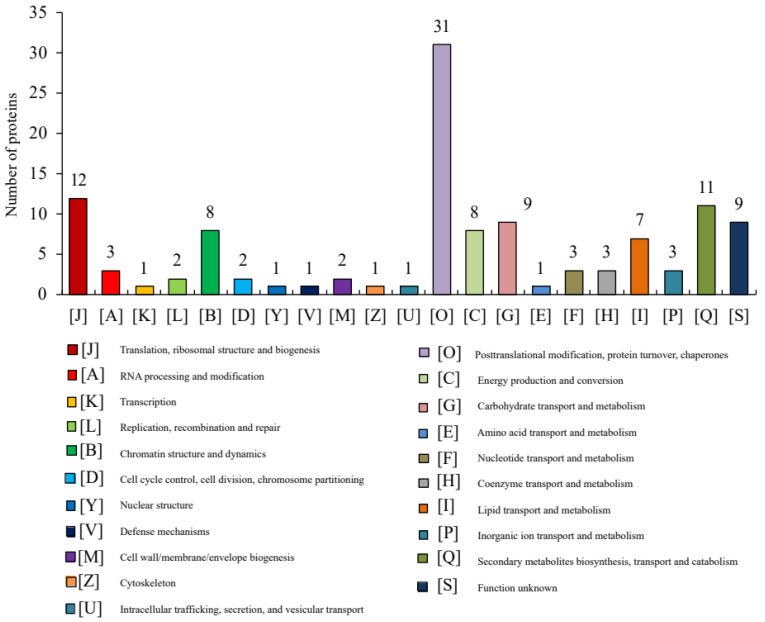
The Clusters of Orthologous Groups (COG) functional annotation of DEPs.

This study primarily focused on the candidate DEPs potentially associated with the differences in drought tolerance between the C105 and E1 varieties. Therefore, only the 169 DEPs identified in the comparison between C_T and E_T were selected for functional annotation. A total of 18 Gene Ontology (GO) terms were significantly annotated at an adjusted *p*-value of < 0.05. Of these, two were classified under the biological process (BP) category, while eight were categorized under both the cellular component (CC) and molecular function (MF) categories (Table S2, Figure 3). In the BP category, DEPs were mostly enriched in response to high light intensity (three DEPs) and negative regulation of gene expression/epigenetic (two DEPs). The CC category saw DEPs primarily enriched in the organelle lumen (22 DEPs), intracellular organelle lumen (22 DEPs), and chromosomes (8 DEPs). In the MF category, DEPs were predominantly enriched in glutathione transferase activity (five DEPs), methionine adenosyltransferase activity (three DEPs), pectinesterase inhibitor activity (three DEPs), and magnesium ion binding (three DEPs). The Clusters of Orthologous Groups (COG) analysis demonstrated that all DEPs could be grouped into 21 functional classes (Table S3, Figure 4). The largest group was “post-translational modification, protein turnover, chaperones”, which included 31 DEPs. The following groups were “translation, ribosomal structure and biogenesis” and “secondary metabolites biosynthesis, transport and catabolism”, comprising of 12 and 11 DEPs, respectively.

**Figure 5:**
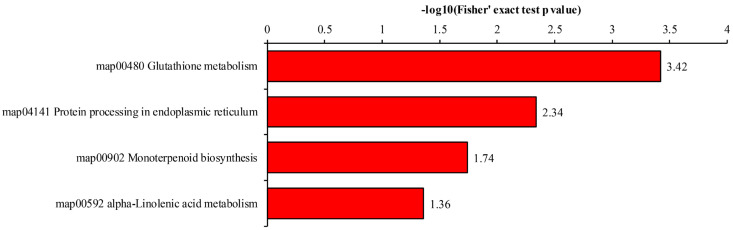
The Kyoto Encyclopedia of Genes and Genomes (KEGG) pathway annotation of DEPs.

The Kyoto Encyclopedia of Genes and Genomes (KEGG) annotation of the DEPs showed that the associated pathways could be divided into four categories at an adjusted *p*-value of < 0.05, that is, glutathione metabolism, protein processing in the endoplasmic reticulum, monoterpenoid biosynthesis, and alpha-linolenic acid metabolism (Figure 5). The most abundant category was protein processing in the endoplasmic reticulum (11 DEPs), followed by glutathione metabolism (8 DEPs). The categories of monoterpenoid biosynthesis and alpha-linolenic acid metabolism contained the fewest proteins (two and three DEPs, respectively).

### 3.4. Protein–Protein Interaction Analysis

**Figure 6:**
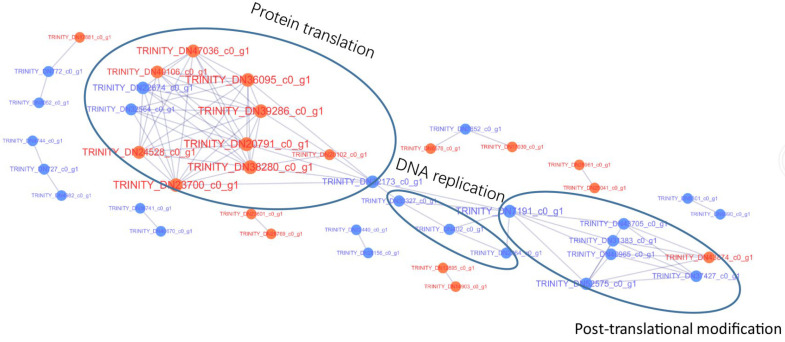
PPI network of DEPs in comparison of C_T and E_T, red represents upregulated differentially expressed proteins, and blue represents downregulated differentially expressed proteins.

Proteins carry out a multitude of functions and often interact with other proteins. Thus, protein–protein interactions (PPIs) are crucial in physiological processes. To predict the relationship among the differentially DEPs, a PPI network was generated using the web-based tool, STRING 9.1. In total, 43 DEPs formed ten interaction groups (Table S4). Among them, 22 DEPs formed three tightly connected functional modules: protein translation, DNA replication, and post-translational modification (Figure 6). The protein translation module mostly comprised a large number of ribosomal proteins, along with some ribosome-binding proteins and translation initiation factors. The DNA replication module was predominantly formed by DEPs dUTP diphosphatase, minichromosome maintenance 2 protein, and kinesin-like protein KIN–10A. The post-translational modification module mostly consisted of histone proteins, which regulate gene expression by modulating the chromatin structure. Interestingly, the accumulation of nine ribosomal proteins increased, whereas the accumulation of other proteins decreased, with the exception of one histone protein (TRINITY_DN43874_c0_g1).

### 3.5. Quantitative Real-Time Polymerase Chain Reaction Assay

**Figure 7:**
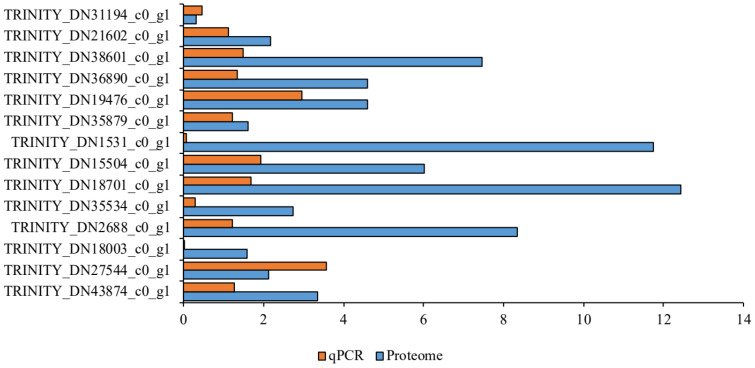
qRT-PCR analysis of the expression correlation between mRNA and DEPs. Three biological repetitions were performed.

The quantitative real-time polymerase chain reaction (qRT-PCR) technique was employed to evaluate the correlation between gene expression levels and proteins. A set of 14 proteins, identified using iTRAQ, were selected for qRT-PCR analyses. These differentially expressed proteins (DEPs) included histone H2B.7-like (TRINITY_DN43874_c0_g1), dehydrin (TRINITY_DN27544_c0_g1, TRINITY_DN19476_c0_g1), THO complex subunit 1 (TRINITY_DN2688_c0_g1), ABA-induced or responsive protein (TRINITY_DN35534_c0_g1, TRINITY_DN35879_c0_g1), late embryogenesis abundant protein (TRINITY_DN18701_c0_g1, TRINITY_DN15504_c0_g1), nitrate regulatory gene2 protein-like (TRINITY_DN1531_c0_g1), chitinase (TRINITY_DN31194_c0_g1), heat shock protein (TRINITY_DN36890_c0_g1), glutaredoxin (TRINITY_DN38601_c0_g1), and DMR6-LIKE OXYGENASE 1 (TRINITY_DN21602_c0_g1). Several of these DEPs have been reported to be involved in responses to drought, such as dehydrin [21], xyloglucan endotransglucosylase [22], ABA-responsive protein [23], late embryogenesis abundant protein [24], and glutaredoxin [25]. Although the transcription and protein abundance of three DEPs (TRINITY_DN18003_c0_g1, TRINITY_DN35534_c0_g1, TRINITY_DN1531_c0_g1) displayed discrepancies (Figure 7), the results were consistent for most DEPs, confirming the reliability of the DEP identification.

### 3.6. Function Validation of the DEPs

The PVX vector allows for high expression of foreign genes, which can then be transferred from the initially inoculated cells to uninfected sites. This makes the PVX vector a valuable tool for silencing or overexpressing genes by ligating fragments or full-length sequences of target genes. Successful application of virus vectors was demonstrated in the investigation of genes involved in drought stress [19].

**Figure 8:**
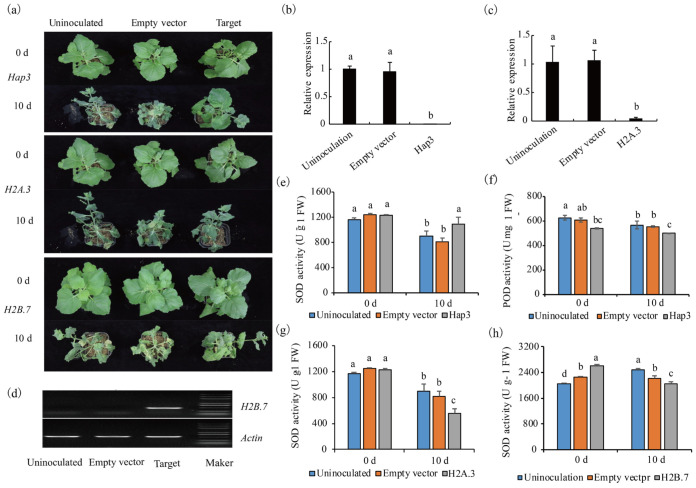
Function validation of *Hap3*, *H2A.3*, and *H2B.7* (**a**) phenotype of plants. (**b**–**d**) The expression of *Hap3*, *H2A.3*, and *H2B.7* detected by qPCR or RT-PCR. (**e**,**f**) Activity of SOD or POD in HAP3-silenced plants and CK. (**g**) Activity of SOD in H2A.3-silenced plants and CK. (**h**) Activity of SOD in H2B.7 ovexpressed plants and CK. The lowercase letters represent significant difference (*p* < 0.05). Values are means ± SE of three replicates.

In this study, three histone family DEPs (TRINITY_DN31383_c0_g1, TRINITY_DN43705_c0_g1, and TRINITY_DN43874_c0_g1), which encode Hap3, H2A.3, and H2B.7, respectively, were selected to validate their function in regulating drought tolerance. These DEPs were chosen because they formed a module of post-translational modification in the PPI analysis (Figure 6). Downregulated differentially DEPs Hap3 and H2A.3 were observed, and their homologues Niben101Scf01372g09016.1 and Niben101Scf05962g04009.1 were subsequently silenced in *N. benthamiana* by ligating their fragments to the PVX vector. Conversely, H2B.7, an upregulated DEP, was overexpressed in *N. benthamiana* through the ligation of its full coding domain sequence to the PVX vector. The silencing of Hap3 and H2A.3, along with the ectopic overexpression of H2B.7, enhanced the drought tolerance of the tobacco plants (Figure 8a). In plants subjected to target silencing or ectopic overexpression, new developing leaves grew normally; however, in control groups, the leaves of either those inoculated with an empty vector or those treated with inoculation solution wilted after ten days of drought stress. In plants where Hap3 and H2A.3 were silenced or H2B.7 was ectopically overexpressed, the expression of Hap3 and H2A.3 was downregulated, whereas H2B.7 expression was upregulated (Figure 8b–d). This suggested that the enhanced drought tolerance was due to either the silencing or overexpression of the respective targets. The activity of superoxide dismutase (SOD) in plants where Hap3 was silenced increased, while the activity of peroxidase (POD) decreased. Conversely, the activities of SOD in plants where H2A.3 was silenced and H2B.7 was overexpressed both showed a decrease compared to that in uninoculated plants or those inoculated with an empty vector under drought stress (Figure 8e–h). However, the activities of POD in plants with silencing of H2A.3 and overexpressed H2B.7, as well as the malondialdehyde (MDA) and proline (PRO) contents in plants with silenced Hap3, H2A.3, or overexpressed H2B.7, did not show significant differences compared to those in uninoculated plants or those inoculated with an empty vector under drought stress (Supplementary Figure S1a–h).

## 4. Discussion

Germination, a transition from a quiescent dry seed to an active metabolic state, is a multifaceted process that is regulated by numerous genes [26]. According to the differences in water absorption during seed germination, seed germination can be divided into three stages. The first stage is the rapid water absorption stage, in which the seeds start to absorb a large amount of water from a dry state and recover to a state where normal physiological metabolism can occur. The second stage is the stage of water absorption stagnation, during which the fresh weight of seeds remains basically unchanged or slightly increases. This stage is also a stage of extremely active physiological metabolism; the third stage is the postgermination stage, during which the seeds germinate by breaking through the seed coat through the embryonic root. In addition, the first two stages of germination are also known as the early stages of germination [27]. In this study, we identified a large number of differentially expressed proteins (DEPs) through the proteomic analysis of drought stress and normal growth treatments during the germination period of drought-tolerant and drought-intolerant seeds (Figure 2). These DEPs primarily function in areas such as “post-translational modification, protein turnover, chaperones” and “translation, ribosomal structure, and biogenesis”, among others. PPI analysis revealed that the majority of DEPs formed interactive groups, with 22 DEPs being closely connected to form functional modules of protein translation, DNA replication, and post-translational modification (Figure 6). This suggests that these DEPs could play significant roles in regulating germination under drought stress conditions.

Ribosomal proteins (RPs) and ribosomal RNA (rRNA), the constituents of ribosomes, function collaboratively in translating the genetic code in messenger RNA (mRNA) and facilitate protein synthesis in all living organisms. The most crucial regulatory target requiring a swift response is the translation activation of mRNAs. Quick translation allows cells to rapidly produce new proteins, enhancing protein output from transcriptionally induced genes but simultaneously reducing the protein output from genes already under transcriptional repression [28]. In this study, nine RPs, namely, S4-1, S15-4, S16, S17, S26-3, L5, L7a-2, L7/L12, and L27, were found to be upregulated in response to drought in the drought-tolerant variety, C105. This indicates that the rate of protein production might increase in C105 under drought stress conditions. It was reported that the activation of the RP genes RPL6 and RPL23A in two rice mutants with high water-use efficiency was triggered by integrated 4× enhancers. The high expression of RPL23A significantly improved fresh weight and root length under drought stress [29]. The eukaryotic translation initiation factor 5A (eIF-5A), a highly conserved repeat factor in eukaryotic organisms, plays a significant role in regulating translation initiation and elongation, mRNA turnover and decay, cell proliferation, and programmed cell death. Overexpression of *Rosa chinensis* RceIF5A enhances resistance to thermotolerance, oxidative stress, and osmotic stress in *Arabidopsis thaliana* [30]. The nascent polypeptide-associated complex (NAC), which binds to the newly emerging nascent polypeptides in ribosomes, is composed of α and β subunits. Knocking down an NAC subunit often increases sensitivity to stress [31]. In this study, two eIF-5A proteins (TRINITY_DN32564_c0_g1 and TRINITY_DN32173_c0_g1) and one NAC were found to be downregulated in C105 compared to E1 under drought stress conditions. This suggests that these negatively regulate the drought response in faba bean.

Three differential DEPs, namely, dUTP diphosphatase (DUT), minichromosome maintenance (MCM) 2 protein, and kinesin-like protein (KLP) KIN-10A, were involved in DNA replication and were identified as a module in PPI. DUT catalyzes the hydrolysis of dUTP to produce precursors necessary for thymidine nucleotides synthesis [32]. KLPs represent a large family of microtubule motor proteins that move in either plus- or minus-end directions. These proteins are involved in intracellular transport, mitosis, meiosis, and cellular development [33]. KLPs have reportedly responded to temperature stresses, such as cold or heat, in banana or rice plants [34,35]. MCM proteins, composed of MCM2-7 subunits, play a crucial role in DNA replication [36]. In our study, DUT, MCM, and KLP were found to be downregulated in the drought-tolerant variety C105 compared to E1 under drought stress. This observation suggests that decreased DNA replication might confer an adaptive advantage to C105 under drought conditions.

The post-translational modification module primarily included histone proteins such as H2A.2, H2A.3, H2B.3, H2B.7, and H4, all of which are involved in modifying the chromatin structure. It is common for plants to adapt to drought stress through transcriptional activation or repression [37]. Changes in chromatin’s structure, mediated by histone modification, are a primary regulatory mechanism. Histones, which include two copies each of H2A, H2B, H3, and H4, modulate the compactness and stability of chromatin by binding to DNA. Drought stress usually induces changes in the acetylation of histones for genes, leading to alterations in genome-wide histone acetylation. Two groups of enzymes determine the levels of acetylated histones: histone acetyltransferases (HATs) increase acetylation by adding acetyl groups to histones, while histone deacetylases (HDACs) reduce acetylation by removing these added acetyl groups. Typically, HATs promote gene activation, and HDACs are associated with gene repression. During drought stress, the expression of HAT or HDAC genes can either increase or decrease [37]. For instance, in rice, genes such as *OsHAG702*, *OsHDA704* were significantly upregulated, while *OsHDA703* and *OsHDA710* were significantly downregulated in response to drought treatment [38]. In our study, the accumulation of six histone proteins decreased while one increased in the drought-tolerant variety C105 compared to E1 under drought stress. These findings suggest that histone-modification-mediated transcriptional activations might be associated with the regulatory responses to drought stress.

The functional validation of three histone proteins demonstrated that the downregulation of Hap3 and H2A.3, as well as the upregulation of H2B.7, could all enhance the drought tolerance of tobacco. This was observed alongside altered levels of superoxide dismutase (SOD) or peroxidase (POD) in transgenic plants subjected to drought stress. These findings imply that histone proteins might modulate genes involved in the regulation of the SOD or POD metabolic pathway, thereby enhancing plant tolerance to drought.

## 5. Conclusions

A series of DEPs were identified in a comparison of the drought-tolerant variety C105 and the drought-sensitive variety E1 under drought stress. The DEPs, which were principally associated with glutathione metabolism and protein processing, were mainly enriched through GO and KEGG analysis. PPI analysis revealed that these 22 DEPs form three interconnected functional modules. These DEPs regulate the drought response by enhancing protein translation, reducing DNA replication, and modifying the chromatin structure. These findings provide basic insights into the genetic regulation of faba beans in response to drought stress during germination and support previous physiological and morphological studies. This study provides useful information for understanding valuable agronomic traits and lays the foundation for cultivating drought-tolerant faba beans during seed germination.

## Data Availability

The original mass spectra can be publicly accessed on iProX (https://www.iprox.cn/, accessed on 1 November 2023) using the subproject ID IPX0007467000.

## Supplementary Materials

The following supporting information can be downloaded at: https://www.mdpi.com/article/10.3390/metabo14030175/s1, Table S1: DEPs in the comparison of C_T_C_CK, E_T_E_CK, C_T_E_T, and C_CK_E_CK; Table S2: Go enrichment of DEPs with significance in the comparison of C_T_E_T; Table S3: KEGG enrichment of the DEPs with significance in the comparison of C_T_E_T; Table S4: DEPs for constructed PPI network; Table S5: Primers used in the present study; Figure S1: Physiological elements of the transgenic and control plants.
