# Supplementary material for: Isobaric Tags for Relative and Absolute Quantitation-Based Proteomics Analysis Revealed Proteins Involved in Drought Response during the Germination Stage in Faba Bean
Source: Metabolites. 2024 Mar 21;14(3):175. doi: 10.3390/metabo14030175 (PMC10971895; doi:10.3390/metabo14030175)
Supplement: Supplementary file 1 [file metabolites-14-00175-s001.zip › Table S5.pdf]

Table S5 Primers used in present study

Gene	Forward primer sequence (5'-3')	Reverse primer sequence (5'-3')
**qRT-PCR**		
TRINITY_DN43874_c0_g1	AGCAGGTTCACCCAGACATTG	GTCTGGAAGATTCAGCAGCAAG
TRINITY_DN27544_c0_g1	TATGGTAACCCTATTAGTGGCGG	GTTGGTGCTTCCATACTCAGGTT
TRINITY_DN19476_c0_g1	TATGGTAACCCTATTAGTGGCGG	TGGTGCTTCCATACTCAGGTTTG
TRINITY_DN2688_c0_g1	GCCATAATACAGCCTGGTCCTC	ACTGAAATGCTGCCGACACG
TRINITY_DN35534_c0_g1	GATACCACTTCAACCGTGCCTC	CTTTGCGTCATCAATGGCTTC
TRINITY_DN35879_c0_g1	CAGCGAAGGCGTAGAAGAAATC	TCTCTCCAGCACTCTCTCCAGC
TRINITY_DN18701_c0_g1	GAATCAAGCCTTAGGACAAACCC	GGAGATACTCACGCCTTGGTTT
TRINITY_DN15504_c0_g1	GGCATCAAATCCACCAAGACAT	TATGTCCCGAACTTCACCAGAG
TRINITY_DN1531_c0_g1	GAGGCTGTATCAACTCGCAACG	GATTCTGAACCTCGCCGTGT
TRINITY_DN31194_c0_g1	CTGTCAAAGCAACTGTCGTGGT	GTTGAACAGTAAGCACTAACAGCGT
TRINITY_DN36890_c0_g1	TTCAACTCGCAACATCCGC	CACTTCTTCCTTCTTCACTCCCG
TRINITY_DN38601_c0_g1	CGGTGGTGAGATTCAAGCAGC	CCAAAACAGAATCACAACCACC
TRINITY_DN21602_c0_g1	CAATCACTTTGTTTCCAAGAGCC	CGAAATCCCTCCAGAACAAGAC
NADHD4	AGGGTTAGTGAGCACCATGC	ATAGCCAAAGGGAATACGCC
**Ectopic expression or silencing**		
H2B.7	CgACgACAAgACCCTATGGCGCCAAAGGGAGAGAAGA	gAggAgAAgAgCCCTTAAGAACTTGTAAACTTGGTG
Hap3	CgACgACAAgACCCTAGGAGCTGAGCAAGCTTTTG	gAggAgAAgAgCCCTGCATGCCAGTAGCACTTGTC
H2A.3	CgACgACAAgACCCTGCGCCAGTTTACCTATCTGC	gAggAgAAgAgCCCTGAACCAACCGATATTGCTG
**RT-PCR**		
HAP3	CCCGCAGTTTTGTTTACAGAT	GCATGCCAGTAGCACTTGTC
H2A.3	CAAAGGTTGTGAATGGGTGA	CTTATCCTTGTTACCAAAAGACCTC
Actin	CCCTCCCACATgCTATTCT	AGAGCCTCCAATCCAGACA
