# Supplementary figures and images for: Isobaric Tags for Relative and Absolute Quantitation-Based Proteomics Analysis Revealed Proteins Involved in Drought Response during the Germination Stage in Faba Bean
Source: Metabolites. 2024 Mar 21;14(3):175. doi: 10.3390/metabo14030175 (PMC10971895; doi:10.3390/metabo14030175)

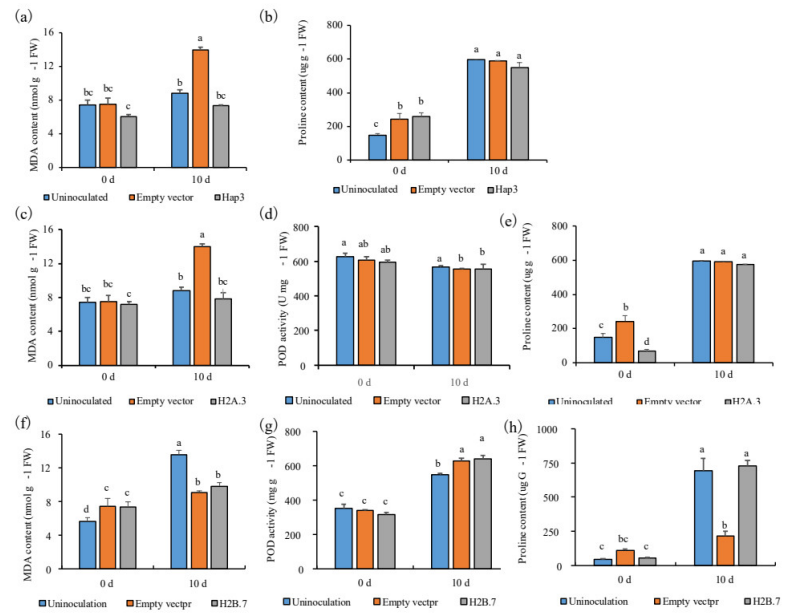

**Figure S1.** Physiological elements of the transgenic and control plants.

Supplement: Supplementary file 1 [file metabolites-14-00175-s001.zip › Figure S1.pdf]
